# Spinal Cord Stimulation in Pediatric Complex Regional Pain Syndrome: A Literature Review

**DOI:** 10.7759/cureus.82581

**Published:** 2025-04-19

**Authors:** Emma E Guld, Katherine R Guld, Tristan Tinney, Mark A Potesta, Kamal Patel

**Affiliations:** 1 Pediatrics, Lake Erie College of Osteopathic Medicine, Bradenton, USA; 2 Emergency Medicine, Lake Erie College of Osteopathic Medicine, Bradenton, USA; 3 Physical Medicine and Rehabilitation, Lake Erie College of Osteopathic Medicine, Bradenton, USA; 4 Internal Medicine, Lake Erie College of Osteopathic Medicine, Bradenton, USA; 5 Pain Management, NeuSpine Institute, Wesley Chapel, USA

**Keywords:** complex regional pain syndrome, complex regional pain syndrome scs, crps scs, crps spinal cord stimulation, crps treatment, pediatric spinal cord stimulation, pediatrics scs, scs, spinal cord stimulation (scs)

## Abstract

Complex regional pain syndrome (CRPS) is a chronic pain condition causing severe pain, sensory disturbances, and functional impairments, disproportionately affecting adolescent females. Spinal cord stimulation (SCS) has emerged as a promising modality for managing refractory CRPS in pediatric patients, offering sustained pain relief and improved quality of life. This literature review evaluates SCS’s efficacy, safety, and functional outcomes in this population, identifies evidence gaps, and provides recommendations for future research. Comprehensive searches were conducted in PubMed, EMBASE, Scopus, Medline, Web of Science, and the Cochrane Central Register of Controlled Trials, including randomized controlled trials, prospective studies, case series, and case reports, focused on pain relief, functional improvement, medication use, and quality of life.

Findings indicate SCS significantly reduces pain, such as Visual Analog Scale scores dropping from 9.2 to 2.9; enhances function, including improved school attendance and physical activity; and decreases medication reliance by 50%-60%, including opioids, with benefits sustained over six months to eight years. Complications, such as cerebrospinal fluid leaks and infections, occur in 16.7% of cases, primarily during trial phases. However, small sample sizes of 7-15 patients, retrospective designs, and a lack of randomized controlled trials limit generalizability. Ethical challenges, including informed consent and device maintenance in growing children, further complicate adoption. Future research should prioritize large-scale, multicenter trials with extended follow-up to confirm long-term outcomes, optimize stimulation parameters, and explore cost-effectiveness. SCS’s success suggests potential applicability to other pediatric pain syndromes, offering an opioid-sparing alternative that addresses both physical and psychosocial dimensions of CRPS, ultimately enhancing well-being in this vulnerable population.

## Introduction and background

Complex regional pain syndrome (CRPS) is a chronic pain disorder characterized by severe pain, sensory abnormalities (e.g., allodynia and hyperalgesia), and autonomic dysfunction (e.g., swelling and color changes), often triggered by minor trauma or nerve injury [1,2]. In children, CRPS has an incidence of 1.14-1.16 per 100,000 annually, predominantly affecting adolescent females [1,2]. It severely impacts physical function, emotional well-being, and social engagement, burdening patients and families [3]. Conventional treatments - physical therapy, cognitive-behavioral therapy, and medications (e.g., antidepressants, antiepileptics, and opioids) - often fail in refractory cases, risking long-term opioid dependency [4,5].

Spinal cord stimulation (SCS) involves implanting electrodes near the spinal cord’s dorsal columns to modulate pain signals, typically following a trial phase to assess efficacy [6]. It has gained traction for managing refractory CRPS in adults, with emerging evidence supporting its pediatric use [6,7]. By altering pain transmission, SCS offers sustained relief and functional restoration. This literature review synthesizes pediatric SCS outcomes, evaluating efficacy, safety, and functional benefits, identifying evidence gaps, and proposing future directions.

## Review

Methods

This literature review involved comprehensive searches in PubMed, EMBASE, Scopus, Medline, Web of Science, and the Cochrane Central Register of Controlled Trials, conducted up to 2024. Search terms included MeSH headings like “spinal cord stimulation,” “complex regional pain syndrome,” and “pediatric,” combined with Boolean operators (e.g., AND, OR). Reference lists of identified studies were hand-searched. Inclusion criteria encompassed studies on SCS in pediatric CRPS patients, reporting outcomes such as pain relief, functional improvement, medication use, or quality of life. Study designs included randomized controlled trials, prospective studies, case series, and case reports. Exclusion criteria covered non-peer-reviewed publications, non-human studies, and those not focused on pediatric CRPS. A total of 20 studies were included.

Pediatric CRPS patients undergoing SCS are primarily adolescent females (aged 11-19), reflecting the condition’s epidemiology [1,2]. Bakr et al.’s series included 12 patients aged 13-19, mostly female, with trauma-related CRPS [7]. Olsson et al. described seven girls aged 11-14 with therapy-resistant CRPS-I, mainly affecting the foot [8]. Abu-Arafeh et al. reported a mean onset age of 13 and a 4:1 female-to-male ratio [1]. Males and upper extremity cases are less common, possibly due to referral bias.

Previous Interventions

Before SCS, patients exhaust conservative options: physical therapy; medications (e.g., gabapentin, amitriptyline, and opioids); cognitive-behavioral therapy; and nerve blocks [9]. Bakr et al. noted an average of 4.9 medications pre-SCS [7]. Olsson et al. reported that all seven patients failed multimodal therapies over months to years [8].

Results

Clinical Outcomes

Pain assessment: SCS consistently reduces pain. Bakr et al. reported a Visual Analog Scale (VAS) drop from 9.2 to 2.9 (p = 0.0002) [7]. Olsson et al. found complete relief in five out of seven patients within two to six weeks, sustained for one to eight years [8]. Stanton-Hicks showed VAS reductions from 8-10 to 2-4 in five patients [4].

Functional outcomes and quality of life: SCS reverses disability. Bakr et al. documented improved mobility and school attendance [7]. Olsson et al. reported that five out of seven patients resumed sports [8]. Karri et al. found that 80% of 15 patients achieved better gait [9]. Pediatric Quality of Life Inventory scores improved from 40 to 75 (p < 0.01) [6]. Klasova et al. noted reduced anxiety (Beck Anxiety Inventory: from 25 to 12) [10].

Medication use: SCS reduces medication reliance. Bakr et al. reported a drop from 4.9 to 2.1 medications (p = 0.0005) [8]. Olsson et al. noted that 50% of patients discontinued analgesics [8]. Karri et al. reported a 60% opioid reduction [9].

Follow-Up Duration

Outcomes are durable. Bakr et al. tracked benefits from six months to five years [6]. Olsson et al.’s one- to eight-year follow-up showed sustained relief in five out of seven patients [8]. Stanton-Hicks reported stability over three to five years [4].

Complication Rates

Complications occur in 16.7% of cases, including cerebrospinal fluid leaks (22% in trials), infections (8%), lead migration, and device malfunction [6]. Olsson et al. noted one infection requiring electrode removal [8]. Pediatric rates may exceed adult norms (3%-6%) due to growth [11].

Stimulation Parameters and Device Placement

Parameters (pulse amplitude: 2-10 mA; width: 200-450 µs; frequency: 40-60 Hz) are tailored [6]. Kriek et al. found high-frequency (500 Hz) settings effective [12]. North et al. emphasized lead placement at T9-T12 for leg pain via a paramedian approach [13]. Ho et al.’s randomized controlled trial showed low-frequency SCS reduced VAS by 3.5 points vs. 1.2 for placebo (p < 0.05) [14].

Discussion

SCS’s efficacy in pediatric CRPS leverages neuroplasticity, achieving a 70% VAS reduction compared to 50%-60% in adults [6,15]. However, evidence is limited by small sample sizes (7-15 patients), retrospective designs, and the absence of large-scale randomized controlled trials [9]. Studies focus on severe cases, potentially overestimating benefits for milder CRPS [7]. SCS’s efficacy reflects its ability to interrupt aberrant pain signaling, leveraging the heightened neuroplasticity of developing nervous systems [16].

Ethical and Practical Considerations

Consent is complex, requiring guardian approval and child assent, balancing surgical risks against uncertain long-term effects [17]. Device maintenance challenges include lead migration and battery replacement in growing spines [11]. Psychosocially, SCS reduces anxiety but may introduce stress from device visibility, necessitating support [10,18].

Comparative Modalities

Dorsal root ganglion (DRG) stimulation offers targeted relief with lower infection rates (2%) but lacks pediatric data [19]. High-frequency or burst SCS may improve outcomes, warranting trials [12]. MRI-compatible devices could reduce revisions [11].

Addressing evidence gaps and limitations

Future research should include multicenter RCTs comparing SCS to sham or DRG stimulation, with 10+ year follow-ups. Ethical frameworks can standardize consent, while larger cohorts clarify outcomes across sexes. Cost-effectiveness studies are needed, as SCS costs ($20,000-$50,000) may offset medication savings [20].

Standardized outcome measures (e.g., VAS vs. PedsQL) are lacking, and short follow-ups (<1 year) in some studies limit durability data [6,9]. Publication bias may favor positive results, and study overlap risks data duplication [6,7].

## Conclusions

SCS significantly reduces pain and improves function in pediatric CRPS, offering an opioid-sparing alternative. Limited by small, retrospective studies and ethical challenges, its adoption requires caution. Large-scale trials and technical refinements are needed to confirm efficacy and optimize use. SCS’s success suggests potential for other pediatric pain syndromes.
